# Supplementary material for: Evolution of Gut Microbiome and Metabolome in Suspected Necrotizing Enterocolitis: A Case-Control Study
Source: J Clin Med. 2020 Jul 17;9(7):2278. doi: 10.3390/jcm9072278 (PMC7408695; doi:10.3390/jcm9072278)
Supplement: Supplementary file 1 [file jcm-09-02278-s001.pdf]

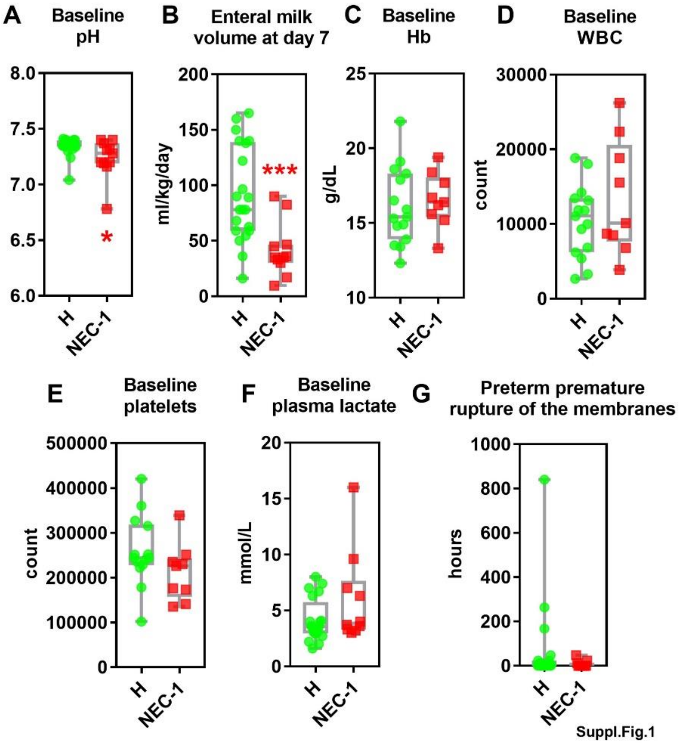

Figure S1. Baseline plasma characteristics in healthy vs. NEC-1 children. A) pH; B) enteral milk volume at day 7; C) hemoglobin (Hb); D) white blood cells (WBC); E) platelets; F) lactate; G) preterm premature rupture of the membranes. *P<0.05, ***P<0.001. Mann-Whitney. N=21 for H and N=11 for NEC-1.

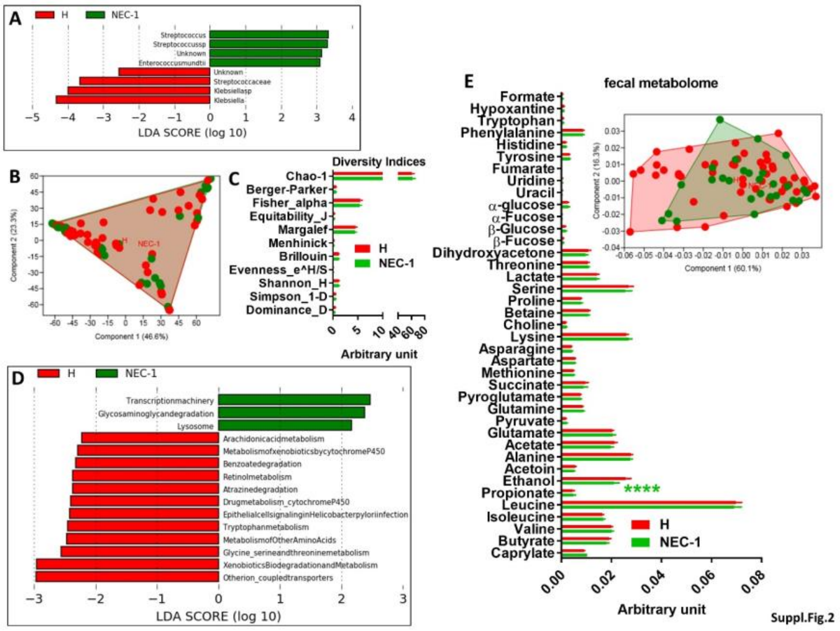

Figure S2. Analysis of gut microbiota, microbiome and metabolome during NEC-1 over the first two months of life. A) Gut microbiota analysis via LDA score between healthy (H) vs. NEC-1 children; B) PCA of the gut microbiota; C) Indices of gut microbiota diversity; D) LDA score for microbial pathways; E) histogram of the overall fecal metabolites and PCA as inset. ****P<0.0001, two-way ANOVA followed by a two-stage linear step-up procedure of Benjamini, Krieger and Yekutieli to correct for multiple comparisons by controlling the False Discovery Rate (<0.05); N=53 for H and N=27 for NEC-1.

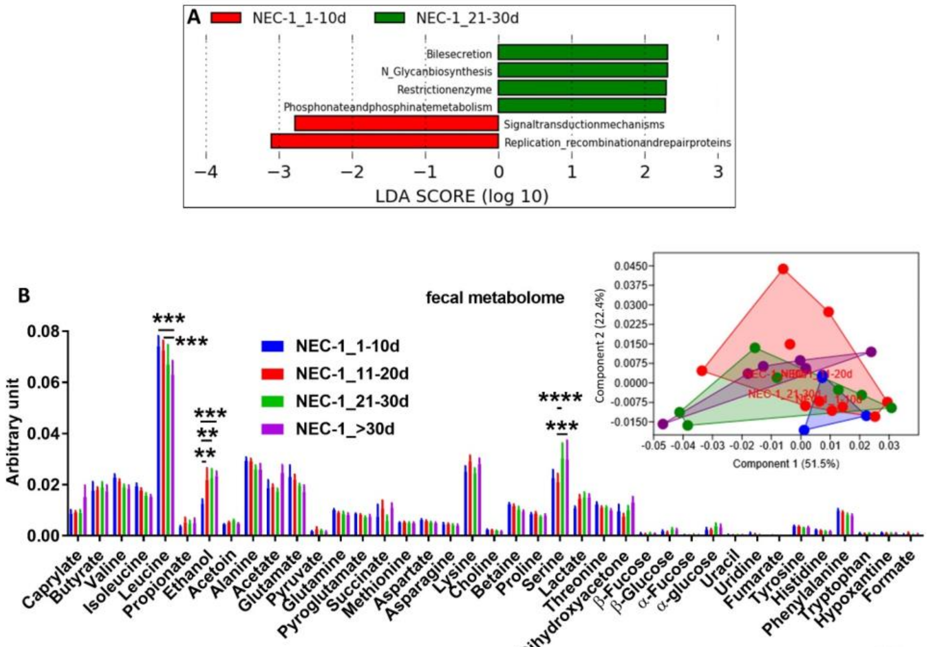

Suppl.Fig.3

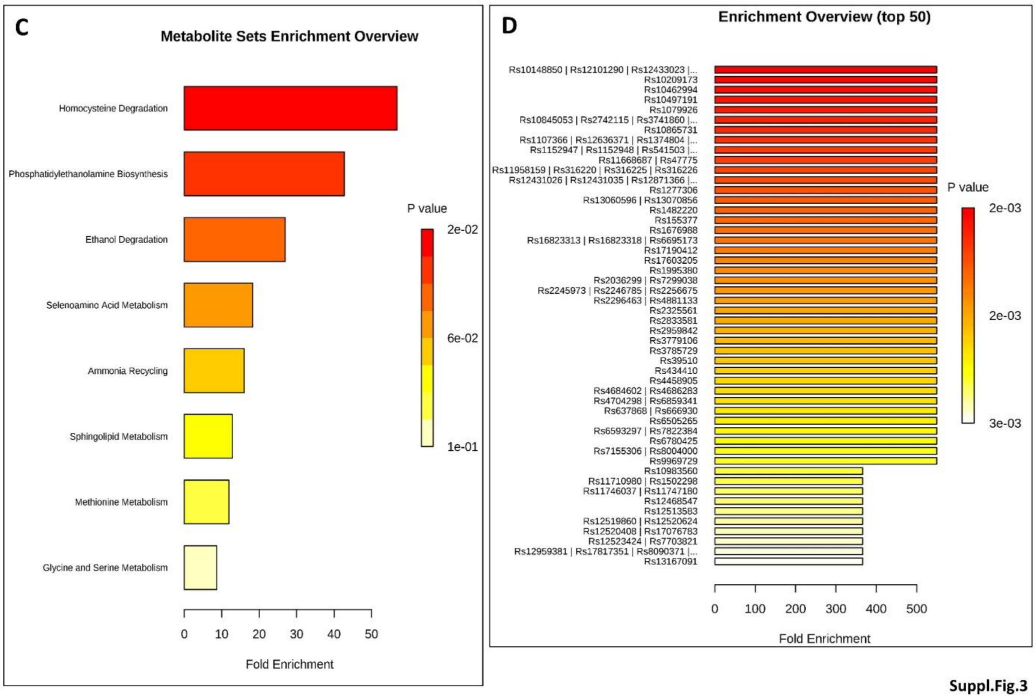

Suppl.Fig.3

Figure S3. A specific microbiome and metabolome exist in healthy vs. NEC-1 children over the first two months of life. A) INTRA_NEC-1 LDA score for microbial pathways; B) histogram of the overall fecal metabolites and PCA as inset; C) INTRA_NEC-1 pathway-associated metabolite sets; D) INTRA_NEC-1 SNP-associated metabolite sets. **P<0.05, ***P<0.01, ****P<0.0001, two-way ANOVA followed by a two-stage linear step-up procedure of Benjamini, Krieger and Yekutieli to correct for multiple comparisons by controlling the False Discovery Rate (<0.05).

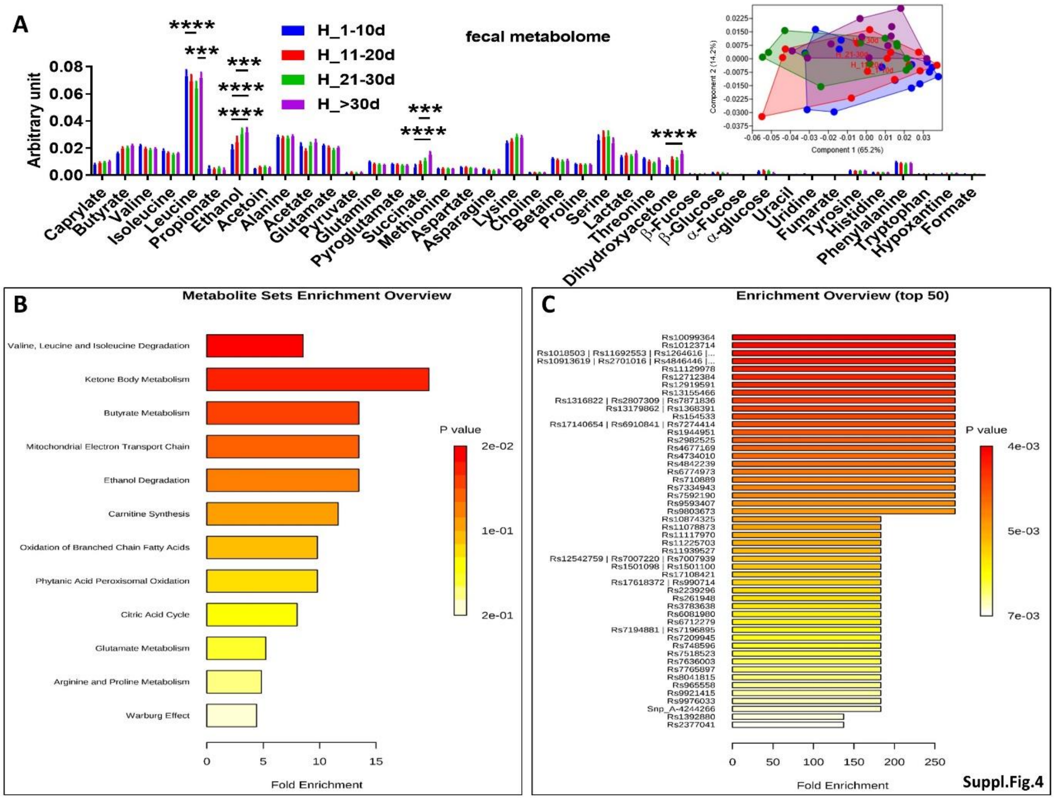

Figure S4. Fecal metabolome progression over the first two months of life in healthy children. A) histogram of the overall fecal metabolites and PCA as inset; B) INTRA_H pathway-associated metabolite sets; C) INTRA_H SNP-associated metabolite sets. ***P<0.01, ****P<0.0001, two-way ANOVA followed by a two-stage linear step-up procedure of Benjamini, Krieger and Yekutieli to correct for multiple comparisons by controlling the False Discovery Rate (<0.05).

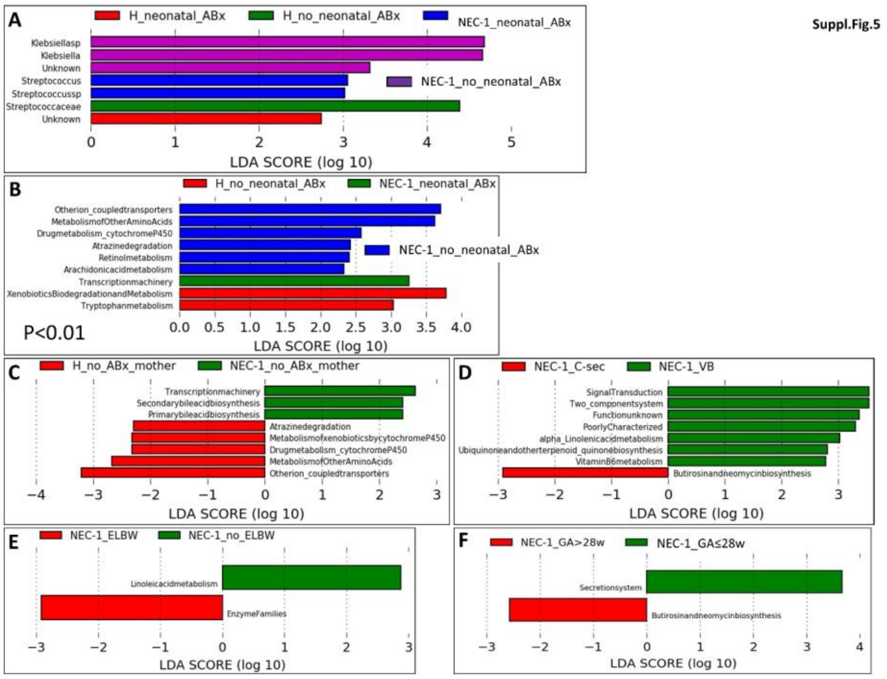

Figure S5. Maternal and child factors shaping gut microbiota and microbiome in healthy vs. NEC-1 children. Six factors were examined: neonatal antibiotics (ABx), ABx treatment on mother, C-section (C-sec) vs. vaginal birth (VB), very low birth weight (VLBW), extremely low birth weight (ELBW) and gestational age (GA) > or ≤ 28 weeks (28w); A) Gut microbiota analysis via LDA score; B) to F) microbiome analysis via LDA score. P<0.05 or P<0.01 as indicated (B).

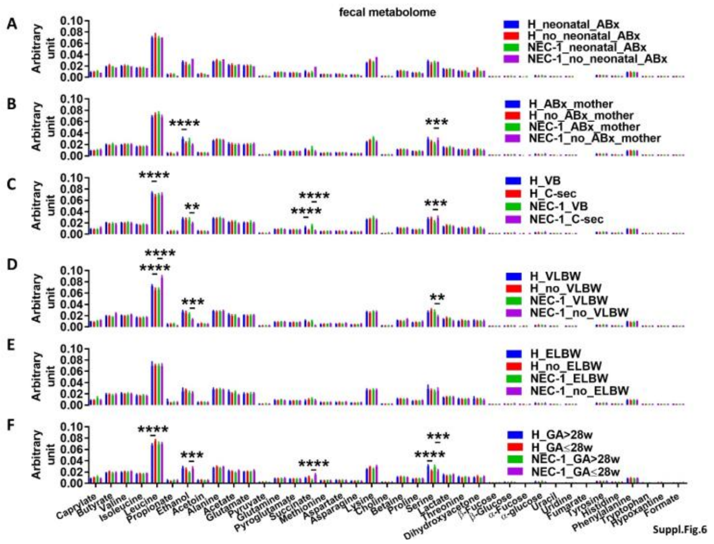

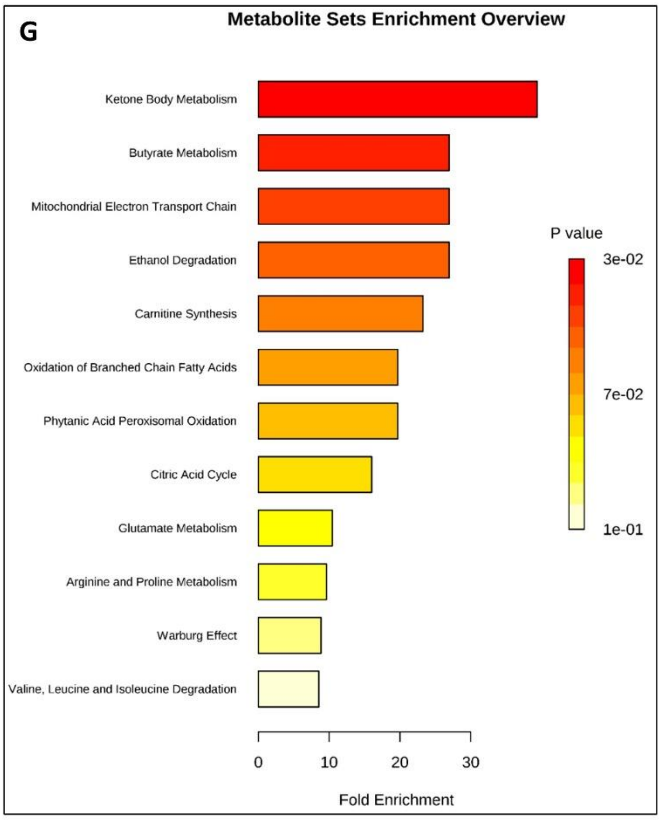

Suppl.Fig.6

Figure S6. Maternal and child factors shaping fecal metabolome in healthy vs. NEC-1 children. Six factors were examined: neonatal antibiotics (ABx), ABx treatment on mother, C-section (C-sec) vs. vaginal birth (VB), very low birth weight (VLBW), extremely low birth weight (ELBW) and gestational age (GA) > or ≤ 28 weeks (28w). A) to F) histogram of the overall fecal metabolites. **P<0.05, ***P<0.01, ****P<0.0001, two-way ANOVA followed by a two-stage linear step-up procedure of Benjamini. Krieger and Yekutieli to correct for multiple comparisons by controlling the False Discovery Rate (<0.05). G) NEC-1_GA≤28w pathway-associated metabolite sets.
